# Methods for identifying the material-recyclable share of SRF during co-processing in the cement industry

**DOI:** 10.1016/j.mex.2020.100837

**Published:** 2020-02-21

**Authors:** Alexia Aldrian, Sandra A. Viczek, Roland Pomberger, Renato Sarc

**Affiliations:** Chair of Waste Processing Technology and Waste Management, Montanuniversitaet Leoben, Franz-Josef-Strasse 18, 8700 Leoben, Austria

**Keywords:** Solid recovered fuel, Recycling, Ash content, Mineral matter, Main components, Methods, Cement industry

## Abstract

Solid Recovered Fuels (SRF) include non-combustible mineral components (e.g. CaCO_3_, SiO_2_, Al_2_O_3_) that are required as raw materials for producing clinker and are completely incorporated into the clinker during the thermal recovery of SRF. This paper discusses simple and practicable ways of finding the relative amount of SRF that may be utilised as raw material (given as the recycling index). For this purpose, the entire mineral content of SRF was determined as the ash content and its main components were identified using different analytical methods.

• A fusion melt of the previously incinerated sample with subsequent measuring using ICP-OES and XRF as well as a total digestion of the incinerated and non-incinerated sample with subsequent measuring using ICP-OES/ICP-MS were applied.
• The results showed a good agreement of all four analytical methods for the elementary oxides Al_2_O_3_, CaO, Fe_2_O_3_, SiO_2_, TiO_2_, P_2_O_5_ and MgO (relative deviation from 6.6 to 38.9%) and slightly higher deviations for K_2_O, Na_2_O and SO_3_ (14.2–96.0%).
• It was also shown that different incineration temperatures (550 °C, 815 °C and 950 °C) have no effect on the result of the recycling index unless it is assumed that the recycling index equals the ash content.

## Graphical abstract

**Figure:**
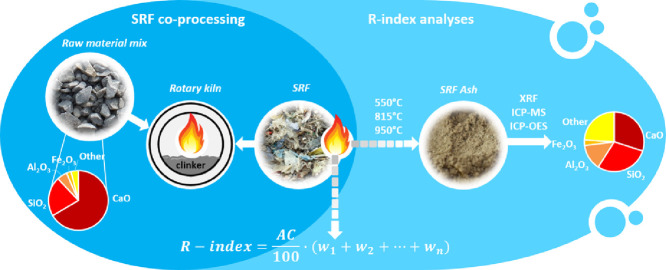

**Table:** Specification Table

Subject Area:	Chemistry
More specific subject area:	*Environmental analytical chemistry*
Method name:	*R-Index*
Name and reference of original method:	*–*
Resource availability:	*–*

## Introduction

Basic raw material components for making cement clinker in a cement plant include calcium oxide (CaO), silicon dioxide (SiO_2_) and small amounts of aluminium oxide (Al_2_O_3_) and iron oxide (Fe_2_O_3_). Available raw materials include limestone, chalk, clay or limestone marl as well as quartz and feldspar, iron hydroxides or iron sulphides. Depending on the raw material deposits at the sites of cement plants, appropriate corrective substances may be required for ideal raw-material mixtures to compensate for missing ingredients. [1]

Cement plants use not only natural raw materials but also secondary raw materials or substitute raw materials. Just like natural raw materials, they contain the main ingredients required for producing cement clinker. There are many arguments for using secondary raw materials: first, natural resources and costs are saved. Moreover, waste that would otherwise have to be landfilled can be persuasively recycled, given that secondary raw materials must not contain any hazardous components that would impair the emissions of the cement plant or the composition of the clinker. [2,3]

Raw and secondary raw materials can be divided into the following groups by composition [3], [4], [5]:

• Ca Group: limestone, marl, chalk, lime sludge from drinking water and sewage treatment, aerated concrete granules, calcium chloride, calcium fluoride, industrial lime waste, carbide sludge, hydrated lime;
• Si Group: sand, foundry sands, silica and quartz waste, sand trap residues, chrome sand, microsilica;
• Fe Group: iron ore, roasted pyrite, contaminated ore, iron oxide/fly ash blends, mill scale, dusts from steel plants, red sludge, converter slag, tin slag;
• Al Group: residues from reprocessing salt slag, aluminium hydroxide, catalyst dust;
• Si-Al Group: clay, bentonites, kaolinites, coal processing residues;
• Si-Al-Ca Group: fly ash, granulated blast furnace slag, oil shale, trass, slag, crushed sand, bleaching earth, aluminium oxide sludge, paper residues, oil contaminated soils, natural stone processing residues.
• S Group: natural gypsum, natural anhydrite, gypsum from flue gas desulfurization;

### Using SRF in the cement industry

Cement clinker production requires a high amount of thermal energy, mainly used for burning. The rather high energy consumption of cement clinker production is fulfilled by traditional fuels such as hard coal, lignite, petroleum coke and, to a lesser extent, petrol oil. Alternative fuels such as Refuse-Derived Fuels (RDF) are also applied. [1] These RDF include hazardous as well as non-hazardous waste like sewage sludge, waste wood, processed fractions of production, household and commercial waste, plastic waste, light shredder fractions, used tires, waste oil and used solvents. Solid Recovered Fuel (SRF) is a subgroup of RDF composed of non-hazardous sorted and mixed solid waste [6], [7], [8], [9]. Two different types of SRF suitable for the use in the cement industry are basically present on the market, classified by their area of application [10,11]:

• SRF for secondary firing (SRF “secondary”): Lower heating value 12–18 MJ/kgOS (corresponding to class NCV 3 or 4 in EN 15,359), grain size <80 mm (used at calciner or kiln inlet) or <300 mm (used for hot disc combustion chamber), respectively.
• SRF for primary firing (SRF “primary”): Lower heating value 18–25 MJ/kgOS (corresponding to class NCV 1, 2, or 3 in EN 15,359), grain size <35 mm (used in primary firing of the rotary kiln of cement plants (main burner fuel)).

The use of waste as substitute raw material or SRF in the cement industry is subject to specific European-based quality requirements defined in the BAT conclusions for the production of cement, lime and magnesium oxide of the European Union [12]. This includes ensuring constant quality as well as defined physical and chemical criteria (e. g. combustibility, reactivity, calorific value, chlorine content, sulphur content). Further aspects of the BAT conclusions concern the application of a quality assurance system for each waste load and control of the amount of relevant parameters such as relevant metals (e. g. cadmium, mercury).

### Composition of SRF

SRF “primary” and SRF “secondary” consist primarily of plastics and of biogenic components. The main fractions are plastic (9.3–42.3 wt%), paper/cardboard/biogenic waste (5.3–25.6 wt%) and textiles (2.1–18.9 wt%). There is also a non-sortable fine fraction (<11.2 mm) (15.4–71.7 wt%) [8,9]. In addition to the combustible fraction, SRF also contain a non-combustible inorganic fraction. This is classified and indicated as ash content. Table 1 presents a selection of the ash contents of SRF samples of different origins given in references. Their range extends from 5.7 wt% to 30.6 wt%.

**Table 1:** Ash content of SRF from different sources (selection) (Abbreviations used: n/s.: not specified; MSW: Municipal solid waste; CW: Commercial waste; IW: Industrial waste; C&DW: Construction and demolition waste).

References	Ash content [wt%]	Incineration temperature	SRF origin
Bourtsalas et al. [13]	10.2–13.8	n/s	MSW
Gallardo et al. [14]	10.7	n/s	MSW
Hilber et al. [15]	7.9	n/s	Mixed waste of MSW and CW
Kara [16]	7.7	n/s	MSW
Kuna [17]	11.1–22.4 Mean: 16.3 (*n* = 3)	815 °C	MSW
Montané et al. [18]	18.2	550 °C	MSW
Nasrullah et al. [19]	12.5	550 °C	CW and IW
Nasrullah et al. [20]	9.0	550 °C	C&DW
Sarc et al. [8]	10.0–19.0 Mean: 14.3 (*n* = 5)	815 °C	SRF “primary”; Mixed waste of MSW and CW
Sarc et al. [8]	13.4 – 26.0 Mean: 17.8 (*n* = 7)	815 °C	SRF “secondary”; Mixed waste of MSW and CW
Sarc et al. [9]	6.3–23.4 Mean: 15.8 (*n* = 13)	815 °C	SRF “primary”; Mixed waste of MSW and CW
Sarc et al. [9]	12.3–30.6 Mean: 20.1 (*n* = 12)	815 °C	SRF “secondary”; Mixed waste of MSW and CW
Velis et al. [21]	17.3	550 °C	MSW
Wagland et al. [22]	11.1 (synthetic SRF) 16.2 (RDF)	n/s	Synthetic SRF: Paper, Pastic, Textile and Wood; RDF: MSW
Wu et al. [23]	12.9	n/s	n/s
Wu et al. [24]	5.7	n/s	n/s

A typical composition of raw meal for the production of Portland cement is 77.36 wt% of CaCO_3_, 13.73 wt% of SiO_2_, 2.93 wt% of Al_2_O_3_, 1.84 wt% of Fe_2_O_3_, 1.83 wt% of MgO, 1.08 wt% of SO_3_, 0.85 wt% of K_2_O, 0.14 wt% of Na_2_O, 0.02 wt% of P_2_O_5_, 0.15 wt% of TiO_2_, 0.06 wt% of Cl and 0.01 wt% of ZnO [25]. All these ingredients are present in the ash residue of SRF. The non-flammable part of SRF is completely integrated into the clinker, i. e. individual components are used for clinker phase formation [1,26]

The chemical composition of the ash is a function of the quality of the SRF, i. e. of its input materials. It is also related to the combustion process and associated conditions (e. g. temperature, availability of oxygen, grain size of SRF used) [27]. Table 2 shows the compositions of SRF ash found in references. The list includes results analysed directly in the ash residues of the SRF.

**Table 2:** Overview of the ash compositions of various SRF (abbreviations used: n/s.: not applicable; MSW: Municipal solid waste; CW: Commercial waste); Values in brackets were calculated from the total sulphur content in the original publication.

Elemental oxide [wt%]	Dunnu et al. [32]	Dunnu et al. [32]	Hilber et al. [15]	Kuna [17]	Pohl et al. [33]	Wagland et al. [22]	Wagland et al. [22]
Al_2_O_3_	11.18	16.18	13.68	12.0–17.3	10.47	4.3	9.5
CaO	25.41	21.80	25.77	20.4–24.5	40.13	60.4	18.5
Fe_2_O_3_	2.88	3.94	3.33	7.0–14.4	4.83	4.5	2.7
K_2_O	2.34	2.82	2.02	1.1–1.8	0.78	0.1	1.9
MgO	3.68	2.59	2.43	2.3–2.8	3.23	1.2	2.0
Na_2_O	4.18	4.80	5.27	2.4–3.8	2.20	0.3	3.3
P_2_O_5_	1.18	1.70	1.26	1.0–1.9	0.51	0.8	1.5
SO_3_	4.50	2.50	1.34	3.6–11.9	(3.42)	(0.17)	(0.81)
SiO_2_	38.12	36.07	26.52	33.4–35.7	23.87	7.5	48.1
TiO_2_	2.33	1.31	2.28	1.6–2.4	2.68	8.1	1.8
SRF origin	MSW	Paper/plastic	MSW/CW	n/s	CW	Synthetic SRF: paper, plastic, textiles, wood	MSW
Determination method	XRF	XRF	n/s	ICP-OES	n/s	ICP-OES	ICP-OES

While the mineralogical composition of coal and coal fly ash is quite well describable due to their natural origin [28], [29], [30], [31], the mineral phases of SRF are much more complex and difficult to classify. This is explained by the fact that the main ingredients of SRF ash do not originate from natural but rather from synthetic products, so that the theoretical range of present mineral phases may be accordingly broad [32]. There is evidence for not only the chemical composition, i.e. the content of Al_2_O_3_, Fe_2_O_3_, CaO, MgO and P_2_O_5_ or the ratio of SiO_2_-Al_2_O_3_ significantly affecting the ash flow temperature but that the mineralogical composition has an impact, too [32]. For example, the melting (temperature difference between shrinkage temperature and flow temperature) of SRF ash occurs over a significantly shorter temperature range (‘short slag’) than in the case of hard coal (‘long slag’). Nevertheless, the ash melting behaviour of SRF shows that SRF ash melts completely during clinker production in the rotary kiln, with flow temperatures in oxidising atmosphere given in sources as 1210 °C [32], 1210–1250 °C [17] and 1300 °C [33], respectively. From a technical point of view, this indicates that complete incorporation of ash into clinker leads to a certain amount of SRF not being thermally recovered but recycled.

### Proposed analytical method for determining the recyclable fraction in SRF

The incombustible fraction of SRF is usually determined as the ash content. The SRF ash consists of a number of components (e. g. SiO_2_, CaO, Fe_2_O_3_) contributing to the raw material content in clinker production. Individual components must be identified analytically. This recyclable fraction of SRF, given as the recycling index (or R-Index), is thus computed according to Formula 1 with w_1_, w_2_, …, w_n_ being those mass fractions of elementary oxides that can be attributed to recycling. The R-Index refers to the dried sample (DM: dry mass).

(1) $$R-Index=\frac{AC}{100}\cdot \left(w_{1}+w_{2}+\ldots +w_{n}\right)$$

*With*

R-Index: Recycling-Index (recyclable fraction in SRF; the reference value is the dried sample) [%_DM_]
AC: Ash content [wt%_DM_]
w _1, 2,__…,__n_: Mass fractions of elementary oxides that can be attributed to recycling [wt%_DM_]

Researched data based on literature references do not permit adequate estimations of the relative amount in SRF that would be attributable to recycling. First, Tables 1 and 2 clearly show that only few data from the references are available for both the ash content and the composition of SRF ashes. Second, quality and reliability of data are insufficient for all parameters due to the large differences between values given in the literature. Furthermore, different incineration temperatures used in determining the ash content (550 °C and 815 °C) and different methods (XRF, ICP-OES) for analysing the ash have been used in the references examined (cf. Tables 1 and 2), preventing direct compatibility of data.

As a first step, the methods for obtaining the fraction attributable to recycling have to be established to define standardised procedures. The objectives of this paper are therefore as follows:

• Investigating suitable methods for establishing the ash content and composition of SRF and comparing them.
• Developing a meaningful, methodical approach for finding the relevant main constituents of ash based on methodical research. Particular attention must be paid to simplicity and easy performance.
• Applying different analysis methods to obtain the ash composition and to compare the results.
• Evaluation of these methods for applicability and suitability.

The final purpose of this paper is to derive a distinct procedure including one or more methods to be used in future for obtaining the ash content and for ash analysis to identify the recyclable fraction of SRF so as to get reliable and representative results.

## Materials and methods

### Survey of relevant standardised methods for the SRF and fuel sector

There are already a number of standardised methods for the analysis of SRF and solid mineral fuels. They include analysis of ash content as well as of its main constituents and are based on international, European and national standards. They have been summarised and compared in Table 3. It clearly follows that while there are some similarities between the analysis of SRF and that of solid fuels, there are also profound differences. The different combustion temperatures applied to analyse the ash content are particularly apparent (550 °C for SRF vs. 815 °C for solid fuels), as are the various options for measuring the main elements. Methods for analysing the ash content and its main elements listed in Table 3 were drawn on for the series of test experiments carried out while compiling this paper.

**Table 3:** Overview on the analytical standard methods for SRF and solid fuels.

Parameters	Solid recovered fuels	Solid fuels
Scope of application	Solid recovered fuels	Solid mineral fuels (e. g. hard coal, coke, lignite, peat, charcoal and briquettes from these materials)
Ash content	**EN 15403**[34] Incineration of the sample at (250 ± 10) °C (60 min) and then at (550 ± 10) °C (120 min) in oxidizing atmosphere until the specified mass constancy is reached. **ISO/CD 21656** (currently under development)	**DIN 51719**[35] Incineration of the sample at (500 ± 10) °C (60 min) and then at (815 ± 10) °C (60 min) in oxidizing atmosphere until the mass remains constant. **ÖNORM G 1074**[36] Incineration of the sample at 500 °C and then at (815 ± 15) °C in oxidizing atmosphere until the mass remains constant. **ISO 1171**[37] Incineration of the sample at 500 °C (60 min) and then at (815 ± 10) °C (60 min) in oxidizing atmosphere until the mass remains constant.
Main components	**EN 15410**[38] Obtaining the mass fractions of the main components according to the following methods: 1) Measurement using e. g. ICP-OES or atomic absorption spectrometry after microwave digestion of the non-incinerated sample with hydrofluoric acid, nitric acid and hydrochloric acid. 2) Measurement using e. g. ICP-OES or atomic absorption spectrometry after digestion of the incinerated sample in a warm water bath with hydrofluoric acid, nitric acid and hydrochloric acid. 3) Measurement using e. g. ICP-OES or atomic absorption spectrometry after digestion of the non-incinerated sample in a furnace with hydrofluoric acid, nitric acid and perchloric acid. 4) Measurement using XRF, with pressed pellets or fused tablets being produced from the samples previously incinerated.	**DIN 51729–10**[39] Obtaining the mass fractions of the main components using XRF after fusion melt (with di-lithium tetraborate, lithium metaborate) of the sample previously incinerated at 950 °C to 1150 °C. **DIN 51729–11**[40] Obtaining the mass fractions of the main components by means of ICP-OES after fusion melt (e. g. with lithium metaborate) of the sample previously incinerated at 1050 °C and dissolution of the fused bead in diluted HCl solution. **DIN 51729–8**[41] Obtaining the sodium and potassium oxide contents by atomic absorption spectrometry or ICP-OES after digestion with hydrofluoric acid and hydrochloric acid of the sample previously incinerated at 1050 °C and dissolution of the fused bead in diluted hydrochloric acid solution. **ISO 13605**[42] Obtaining the mass fractions of the main components using XRF after fusion melt (with di-lithium tetraborate, lithium metaborate) of the sample previously incinerated at 815 °C.

### Description and preparation of samples

Altogether, 80 real SRF samples were available for the series of experimental tests. Various samples were randomly selected for the test series from these 80 samples.

The SRF samples were provided by various SRF manufacturers and cement plants in four European countries (Austria, Slovenia, Croatia and Slovakia). The samples were taken by staff of the recycling plants or cement plants or by staff of the Chair of Waste Processing Technology and Waste Management, pursuant to the requirements of EN 15442 [43]. The samples all originated from the production years 2018 and 2019. A total of 50 samples of SRF “primary” and 30 samples of SRF “secondary” were available.

Pursuant to EN 14346 [44], original SRF samples were dried in a drying oven at 105 °C till mass constancy - note: comparable content and same requirement, i.e. 105 °C, as defined in ONR CEN/TS 15414-1 too [45] - and then comminuted to <0.5 mm using a fast-rotating cutting mill (Fritsch, Pulverisette 18 with cyclone) pursuant to the specifications of EN 15413 [46]. The samples were then again dried in a drying oven at 105 °C before incineration or melting.

In some cases, stored samples (already prepared to < 0.5 mm and dried) were provided by some companies. These samples were dried in a drying oven at 105 °C before incineration or melting.

### Analytical methods for finding the main mineral components

**Fig. 1:**
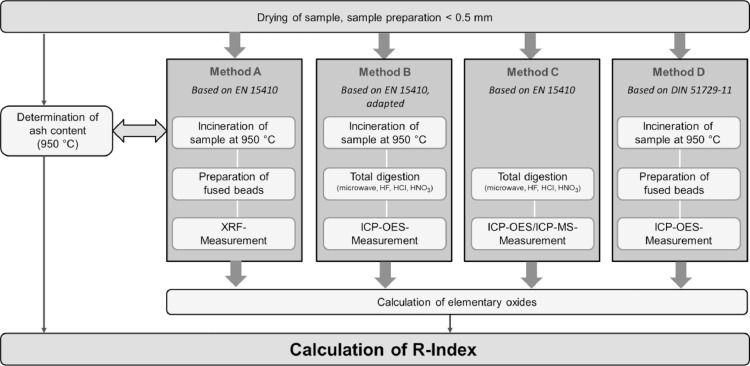
Overview on the different analytical approaches for the determination of the R-Index.

A detailed representation of the individual processing stages is shown in Fig. 1 for all analytical methods used. The major element contents of the mineral substance were analysed for some randomly selected samples (Primary 2, 3, 4, 5, 6 and Secondary 17, 19, 20) for comparisons of all four analytical methods.

The methods applied are standard procedures, some of which having been modified. Incinerated residue was used for methods A, B and D, the dried and prepared sample was used for method C. The sample comminuted < 0.5 mm and dried at 105 °C was incinerated in a muffle furnace (Nabertherm L 9) at a temperature of (950 °C ± 25) °C for a period of 2 h, pursuant to the specifications of EN 196-2 [47]. EN 196-2 is a commonly used standard in the cement industry for the preparation of samples for XRF analyses.

The relative amounts of the main elements aluminium (Al), calcium (Ca), iron (Fe), potassium (K), magnesium (Mg), sodium (Na), phosphorus (P), sulphur (S), silicon (Si) and titanium (Ti) were obtained for all analytical methods since they were identified as main components in the SRF ashes by X-ray fluorescence analysis (see method A). Analysed samples also included the elements Cr, Mn, Ba, Sr, Cu and Zn in measurable concentrations. The relative amounts of these elements, however, were all very low for the investigated samples (Cr_2_O_3_ < 0.1 wt%, MnO < 0.2 wt%, BaO < 0.4 wt%, SrO < 0.06 wt%, Cu < 0.5 wt% Zn < 0.4 wt%). The contents of all other elements (e. g. V, Co, Ni, Cu, Zn, Pb) were below 0.05 wt%. For this reason, only the main elements were included in further measurements.

**Method A:** For measurements using X-ray fluorescence analysis (XRF), fused beads were made out of SRF samples incinerated at 950 °C. In each case, 1 g of sample was thoroughly mixed with 8 g of di-lithium tetraborate (Li_2_B_4_O_7_, Sigma Aldrich) and melted in a platinum crucible (HD Elektronik und Elektrotechnik GmbH, Fusion Machine Type VAA2). The fused bead was then measured using XRF (PANalytical, Axios). The software GeoWSU was used for quantitative evaluation. This procedure is pursuant to Section 10.3 of EN 15410 [38]. Examinations were carried out in duplicate.

**Method B:** Samples incinerated at 950 °C were digested pursuant to EN 13656 (ASI, 2002) and the main elements subsequently measured using ICP-OES. 0.2 g of the incinerated samples were weighed and 6 ml of hydrochloric acid (HCl), 2 ml of nitric acid (HNO_3_) and 2 ml of hydrofluoric acid (HF) added before heating the samples in a microwave oven (MLS, Ethos). Next, the HF was complexed with boric acid. The digestion solution was made up to a final volume of 50 ml with deionised water (< 0.055 µmS/cm) and measured using ICP-OES (Varian Vista-MPX CCD Simultaneous, Software: 4.1.0) at the following wavelengths: Al 308.215 nm; Ca 317.933 nm; Fe 238.204 nm; K 766.491 nm; Mg 279.553 nm; Na 589.592 nm; P 213.618 nm; S 180.669 nm; Si 251.611 nm; Ti 334.941 nm. The measured element contents were then converted into the respective oxides (Al_2_O_3_, CaO, Fe_2_O_3_, K_2_O, MgO, Na_2_O, P_2_O_5_, SO_3_, SiO_2_ und TiO_2_) Examinations were carried out in duplicate. This approach corresponds in large parts to EN 15,410 (Section 9.1) but was slightly adapted.

**Method C:** This procedure corresponds to Section 9.1 for the identification of the main elements in SRF samples described in EN 15410 [38]. The dried sample, prepared to < 0.5 mm but not incinerated, was decomposed pursuant to EN 13656 [48]. 0.2 g were weighed and 6 ml of hydrochloric acid (HCl), 2 ml of nitric acid (HNO_3_) and 2 ml of hydrofluoric acid (HF) were added before heating the samples in a microwave oven (MLS, Ethos). Next, the HF was complexed with boric acid. The digestion solution was made up to a final volume of 50 ml with deionised water (< 0.055 µmS/cm). The main elements were then measured using ICP-OES and ICP-MS. The elements Al, Ca, K, Mg, Na, P, S, Si and Ti were measured using ICP-OES (Varian Vista-MPX CCD Simultaneous, Software: 4.1.0; see Method B) and the element Fe was measured using ICP-MS (Agilent, 7500ce; due to matrix effects in ICP-OES). The measured element contents were then converted into the respective oxides. Examinations were carried out in duplicate. In method C, oxide contents were obtained that are not related to the ash but to the dry original sample. Results were therefore compared with the relative ash content at 950 °C (cf. Table 6) of the respective sample so that they could be directly compared with the results of other methods.

**Method D:** The procedure complies with what is described in DIN 51729–11 [40] for identifying the main elements in solid fuels. 0.1 g of the incinerated samples were thoroughly mixed with 1 g of melting reagent (lithium metaborate, Sigma Aldrich) in a platinum crucible and melted in a muffle furnace (Nabertherm L 9) at 1050 °C for 20 min. The resulting fused bead was then cooled and dissolved in doses with a total of 80 ml of hydrochloric acid (*c* = 2 mol/l) while heating (at approx. 50 - 60 °C) and stirring (PTFE stirring bone). The solution was filled to a final volume of 250 ml with deionised water (< 0.055 µmS/cm). The digestion solutions were measured using ICP-OES (Varian Vista-MPX CCD Simultaneous, software: 4.1.0) at the wavelengths given for Method B. The measured element contents were again converted into the respective oxides. The limit of determination for the analysis procedure was 0.2 wt% for Al_2_O_3_, CaO, Fe_2_O_3_, K_2_O, MgO, Na_2_O, TiO_2_ and 0.3 wt% for P_2_O_5_, SO_3_ and SiO_2_. All experimental tests were carried out once.

For method D, accuracy and precision of the analytical method applied to SRF were also measured since the standard has actually been developed for solid fuels and not solid recovered fuels. Regarding trueness, proficiency testing materials 1, 2 and 3 of the German supplier DCC (Delta Coal Control) from the years 2016, 2017 and 2018 were measured. To establish repeatability, four randomly selected SRF samples (Primary 21, 24 and 39 as well as Secondary 1) were each measured ten times using the previously described procedure. For the samples, ten separate experiments were carried out including the following steps: incineration, digestion and ICP-OES measurement under repeatable conditions (in each case the same experimenter, the same instruments).

### Effects of incineration temperatures on the R-Index

For 22 selected SRF samples (Primary 1, 3, 4, 5, 6, 7, 19, 20, 22, 27, 32, 36, 40, 41, 44 and Secondary 17, 18, 19, 20, 21, 24, 28), effects of incineration temperatures on obtaining the R-Index was examined. These samples (< 0.5 mm and dried) were incinerated in a muffle furnace (Nabertherm L 9) at 950 °C for 2 h (see Section 2.3). They were also incinerated at 550 °C pursuant to EN 15403 (1 h 550 °*C* ± 10 °C) [34] and at 815 °C according to DIN 51719 (2 h at 500 °C ± 10 °C, then 1 h at 815 °C ± 10 °C) [35] using the muffle furnace. Ash residues obtained at different temperatures were examined for the main components following Method D.

All ash residues were also examined for their carbonate content (as TIC), identified using a Scheibler apparatus according to ÖNORM L 1084 [49]. In each case, diluted hydrochloric acid was added to the incinerated sample to digest the carbonates. The resulting carbon dioxide (CO_2_) was measured using gas volumetry, taking air pressure and temperature into account.

## Results and discussion

### Trueness and precision of method D

The range of application provided in the standard DIN 51729–11 [40] is limited to solid fuels, SRF are not actually covered. Trueness and precision of the standardised procedure applied to SRF were therefore verified. The proficiency testing materials provider DCC (Delta Coal Control) provides interlaboratory comparison participants with various solid fuel samples, including SRF. Parameters analysed in the years 2016, 2017 and 2018 included the identification of the main components pursuant to EN 15410. A comparison of measurement results obtained using method D (incinerated sample, fused beads, ICP-OES) with those of participants in the interlaboratory comparison (mean values after eliminating outliers, known as assigned values) [50], [51], [52] showed very good agreement. Table A1 (cf. Appendices) gives the results measured using method D for the selected elementary oxides for three proficiency testing materials including the calculated z-scores. Z-scores are a common evaluation tool for interlaboratory comparisons and are computed as follows:

(2) $$z-Score=\frac{\left(Result-Assigned\;value\right)}{Variance}$$

A z-score of < 2 gives excellent agreement of the participant result with the assigned value. A z-score of 2–3 is satisfactory and a z-score of > 3 is not satisfactory. Results obtained from analysing proficiency testing materials using method D show z-scores < 2 and very good agreement with the assigned values (cf. Table A1).

Table 4 gives the repeatability results for four randomly selected SRF samples (Primary 21, 24 and 39 and Secondary 1) for Method D. The table includes results for all 10 samples of each material obtained under repeatable conditions (same experimenter, same instruments) as well as arithmetic mean, standard deviation and relative standard deviation (RSD).

**Table 4:** Precision of the analytical approach for four SRFs (Primary 21, 39; Secondary 1, 24) in comparison with the relative standard deviation given in EN 15410.

Sample Identification	Al_2_O_3_ [wt%]	CaO [wt%]	Fe_2_O_3_ [wt%]	K_2_O [wt%]	MgO [wt%]	Na_2_O [wt%]	P_2_O_5_ [wt%]	SO_3_ [wt%]	SiO_2_ [wt%]	TiO_2_ [wt%]	SUM [wt%]
**RSD [%] ÖNORM EN 15410 [****38****]**	**5/32**	**2/15**	**4/24**	**9/10**	**16/135**	**10**	**4/15**	**–**	**2/16**	**2/23**	**–**
Primary 21/1	43.4	20.6	1.0	0.3	1.0	0.4	0.2	2.6	10.8	2.0	82.4
Primary 21/2	43.7	21.5	1.0	0.3	1.1	0.4	0.3	3.2	10.4	2.1	83.9
Primary 21/3	41.0	26.5	1.1	0.2	1.1	0.4	0.3	3.8	11.1	2.3	87.7
Primary 21/4	41.0	23.4	1.4	0.3	1.1	0.4	0.3	3.7	11.1	2.1	84.7
Primary 21/5	39.0	25.1	1.0	0.2	1.2	0.3	0.3	3.2	10.0	2.3	82.5
Primary 21/6	39.3	22.6	1.1	0.2	1.1	0.4	0.3	3.2	10.0	2.3	80.5
Primary 21/7	42.5	21.5	1.2	0.2	1.1	0.4	0.3	3.0	10.8	2.1	83.1
Primary 21/8	41.6	24.5	1.1	0.2	1.1	0.4	0.2	3.8	11.2	2.1	86.1
Primary 21/9	43.3	21.4	1.2	0.3	1.1	0.5	0.3	3.9	10.5	2.2	84.6
Primary 21/10	41.8	22.9	1.0	0.3	1.1	0.4	0.3	3.6	10.1	2.2	83.8
**Mean**	**41.6**	**23.0**	**1.1**	**0.3**	**1.1**	**0.4**	**0.3**	**3.4**	**10.6**	**2.2**	**83.9**
**Standard deviation**	**1.6**	**1.9**	**0.1**	**0.0**	**0.0**	**0.1**	**0.0**	**0.4**	**0.5**	**0.1**	**2.0**
**RSD [%]**	**3.9**	**8.2**	**11.7**	**16.0**	**3.9**	**14.8**	**8.9**	**12.3**	**4.3**	**4.7**	**2.4**
Primary 39/1	28.0	27.0	2.6	0.8	2.3	1.2	0.8	2.6	27.0	6.4	98.8
Primary 39/2	29.3	28.5	2.1	0.7	1.9	0.9	0.4	2.3	27.2	6.4	99.7
Primary 39/3	32.2	26.9	2.7	0.9	1.7	0.7	0.5	1.9	25.2	6.4	99.1
Primary 39/4	29.1	30.2	3.3	1.0	1.8	0.9	0.5	2.3	24.0	6.8	100.0
Primary 39/5	25.0	29.2	2.5	0.8	1.8	1.0	0.6	2.7	26.0	5.4	95.0
Primary 39/6	29.0	26.1	2.0	0.6	1.8	1.0	0.5	1.7	25.2	5.5	93.3
Primary 39/7	27.1	25.3	3.2	0.7	1.7	0.9	0.5	1.5	24.5	5.6	91.1
Primary 39/8	29.8	27.2	2.2	0.9	1.9	1.1	0.5	2.0	26.2	5.6	97.4
Primary 39/9	30.0	24.7	2.2	1.0	1.7	1.0	0.4	1.7	26.4	6.0	95.2
Primary 39/10	27.5	26.6	2.3	0.8	1.8	1.1	0.5	2.1	25.7	5.6	93.9
**Mean**	**28.7**	**27.2**	**2.5**	**0.8**	**1.8**	**1.0**	**0.5**	**2.1**	**25.7**	**6.0**	**96.3**
**Standard deviation**	**1.9**	**1.7**	**0.5**	**0.1**	**0.2**	**0.1**	**0.1**	**0.4**	**1.0**	**0.5**	**3.1**
**RSD [%]**	**6.8**	**6.3**	**18.1**	**16.6**	**10.2**	**14.2**	**20.0**	**18.5**	**4.0**	**8.2**	**3.2**
Secondary 1/1	6.6	26.6	5.0	2.7	3.1	2.8	1.5	5.6	33.7	4.5	92.2
Secondary 1/2	6.9	26.9	4.9	2.6	3.2	2.5	1.6	5.6	33.2	5.0	92.5
Secondary 1/3	7.0	26.5	4.7	2.5	3.0	2.5	1.6	5.8	33.8	4.3	91.7
Secondary 1/4	6.6	27.2	4.4	2.6	3.0	2.1	1.6	6.6	32.4	4.7	91.0
Secondary 1/5	6.4	25.8	4.4	2.3	3.1	2.7	1.6	5.3	33.2	4.3	89.1
Secondary 1/6	6.6	26.5	4.6	2.6	3.0	2.5	1.5	6.6	33.1	4.4	91.2
Secondary 1/7	7.1	26.7	4.6	2.5	3.2	2.6	1.5	5.0	33.7	3.9	90.7
Secondary 1/8	6.6	27.3	6.4	2.5	3.1	2.2	1.5	5.3	32.0	4.5	91.5
Secondary 1/9	6.4	26.6	4.5	2.9	3.0	2.7	1.5	5.3	32.3	4.5	89.8
Secondary 1/10	6.3	25.7	4.9	2.5	3.0	2.2	1.5	7.1	32.8	4.1	90.1
**Mean**	**6.6**	**26.6**	**4.8**	**2.6**	**3.1**	**2.5**	**1.5**	**5.8**	**33.0**	**4.4**	**91.0**
**Standard deviation**	**0.3**	**0.5**	**0.6**	**0.1**	**0.1**	**0.2**	**0.1**	**0.7**	**0.6**	**0.3**	**1.1**
**RSD [%]**	**3.8**	**2.0**	**12.5**	**5.7**	**2.3**	**9.9**	**4.0**	**11.7**	**1.9**	**6.8**	**1.2**
Secondary 24/1	10.3	26.3	4.6	1.6	3.7	1.5	0.7	4.9	39.6	1.3	94.5
Secondary 24/2	11.5	24.9	4.8	1.8	3.6	1.7	0.6	4.3	39.6	1.1	93.8
Secondary 24/3	8.6	26.4	4.3	1.9	3.8	1.9	0.7	5.1	37.7	1.1	91.6
Secondary 24/4	9.4	23.3	5.2	1.9	3.5	1.9	0.7	4.4	40.9	1.3	92.6
Secondary 24/5	9.6	25.7	4.6	1.7	3.9	1.7	0.7	5.8	34.5	1.3	89.7
Secondary 24/6	9.5	27.9	5.2	1.9	3.9	1.7	0.7	4.7	37.6	1.3	94.4
Secondary 24/7	11.0	21.9	5.2	1.7	3.9	1.5	0.7	5.5	39.8	1.5	92.7
Secondary 24/8	11.1	20.0	4.6	2.3	3.5	2.2	0.6	4.0	43.9	1.2	93.3
Secondary 24/9	9.6	25.9	5.5	1.8	3.0	1.6	0.6	3.3	36.9	1.1	89.3
Secondary 24/10	11.6	21.1	4.5	2.2	3.7	1.8	0.6	5.0	42.0	1.3	93.6
**Mean**	**10.2**	**24.3**	**4.8**	**1.9**	**3.7**	**1.7**	**0.7**	**4.7**	**39.3**	**1.2**	**92.6**
**Standard deviation**	**1.0**	**2.6**	**0.4**	**0.2**	**0.3**	**0.2**	**0.1**	**0.7**	**2.7**	**0.1**	**1.8**
**RSD [%]**	**10.0**	**10.8**	**8.1**	**11.9**	**7.5**	**11.7**	**7.8**	**15.6**	**6.8**	**9.2**	**2.0**

EN 15410 [38] (Annex B) includes the characteristic process data for all elements except for sulphur. Accurate precision (the coefficient of variation of the precision) of the standard for SRF (made from municipal waste) is also given in Table 4.

The precision specified in EN 15410 [38] for SRF using method D is certainly achieved and in many cases undercut. The only exceptions are potassium and sodium, two samples of which showing slightly higher precision. But the results are only up to 16.6% and can therefore be regarded as satisfactory for a multistage analytical process.

### Comparing the results of the four analytical methods applied to obtain the main mineral elements

**Fig. A1:**
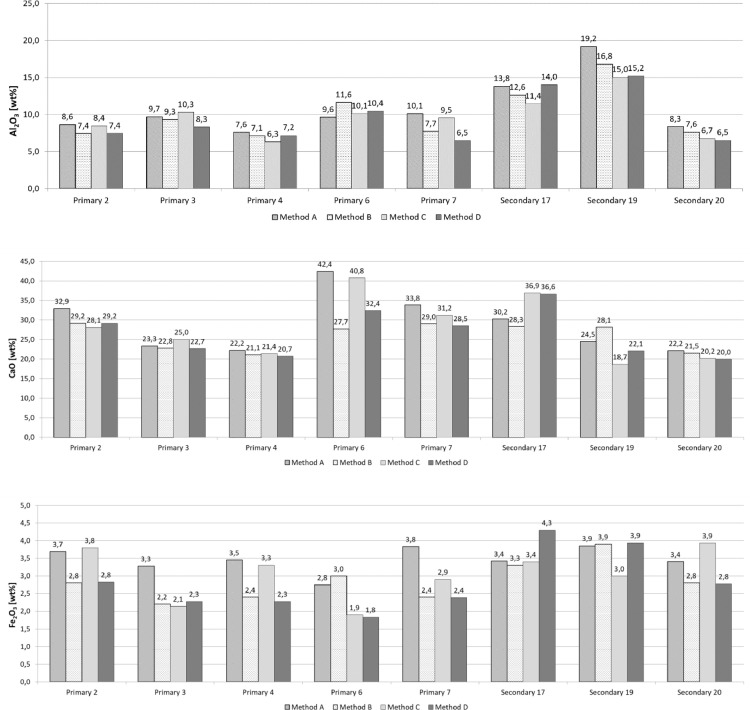
Results for Al_2_O_3_, CaO and Fe_2_O_3_ and for all analytical methods for randomly selected samples.

**Fig. A2:**
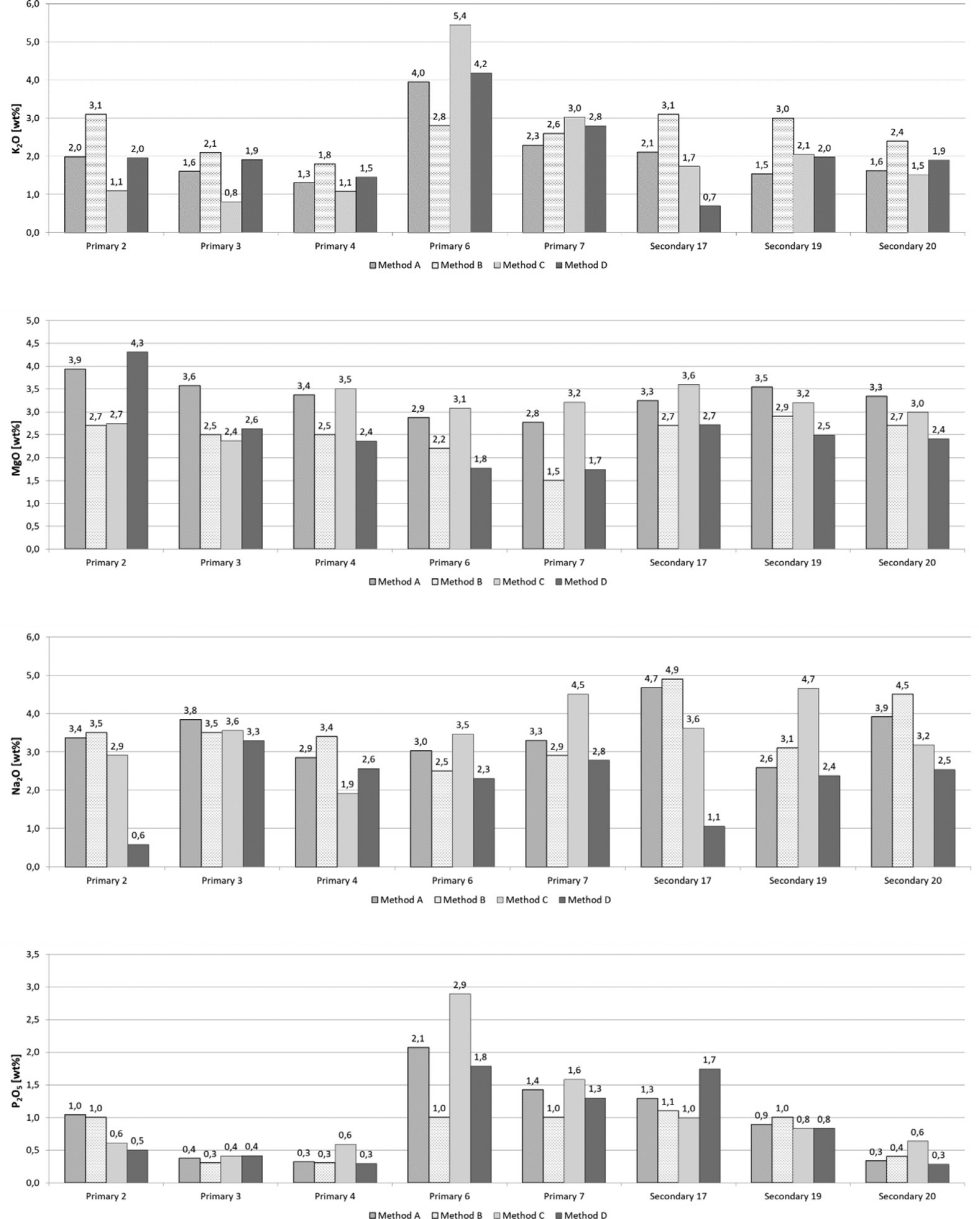
Results for K_2_O MgO, Na_2_O and P_2_O_5_ and for all analytical methods for randomly selected samples.

**Fig. A3:**
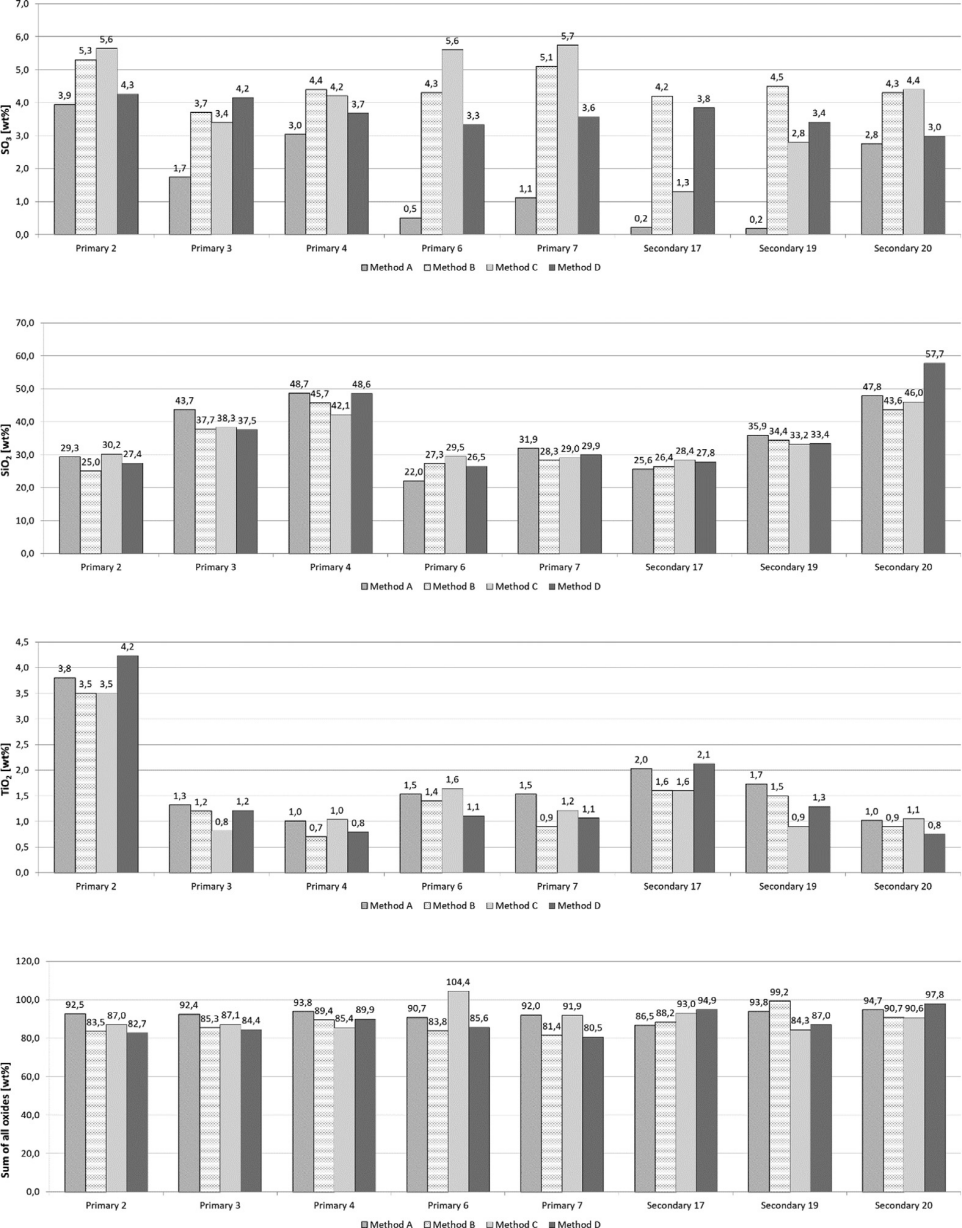
Results for SO_3_, SiO_2_, TiO_2_ and the sum of all measured oxides for all analytical methods for randomly selected samples.

Selected SRF samples (Primary 2, 3, 4, 5, 6 and Secondary 17, 19, 20) were analysed applying all four analytical methods (Method A, B, C and D). Figs. A1–A3 (cf. Appendices) show the results of the four analytical methods for all elementary oxides compared. Methods A, B and C were each carried out twice, with the duplicates matching perfectly (relative deviations <10%). The only exception is method C whose relative deviations between the duplicate analyses were partly >30%. Each of the results shown in the illustrations indicates the mean value of these duplicates.

The total of all measured and analysed elementary oxides for all four methods resulted in values between 80.5 and 99.2 wt%, with one exception (104.4% for Primary 6, Method C) (cf. Fig. A3). As an average (arithmetic mean), the following results for the total of all measured elements were obtained for the eight analysed samples:

• Method A: 91.9 wt%,
• Method B: 87.5 wt%,
• Method C: 90.1 wt% and
• Method D: 87.9 wt%.

Obviously the results obtained by RFA (Method A) tend to be slightly higher (the total of all measured elements was between 86.5 and 94.7 wt%). Theory suggests that the total of all elementary oxides should be close to 100 wt%, therefore the achieved values may be interpreted as satisfying when applying good laboratory practice. Deviations from 100 wt% may on the one hand be due to inherent measurement inaccuracies of the respective analytical method. On the other hand, note that not all components of the mineral content were identified because measurements were limited to elementary oxides identified as main constituents.

In Table 5, the highest and lowest results (minimum and maximum) of all four methods are shown for each elementary oxide and each sample analysed. Relative deviations (in %) between the highest and lowest results are also shown in this table, ranging from 6.6 to 96.0%. The relative deviations for the elementary oxides Al_2_O_3_, CaO, Fe_2_O_3_ and SiO_2_ are <40% (6.6 to 38.9%). Basically this indicates a very good agreement between the various analytical methods. Although a relative deviation of about 40% between analytical methods may at first glance appear to be significant, note that each method comprises a number of steps, all of which can be flawed. The emerging maximum error estimation is called extended inaccuracy in analytics. For method D, this extended inaccuracy has been assessed compliant with the EURACHEM/CITAC guidelines [53]: 32% Al_2_O_3_, 34% CaO, 45% Fe_2_O_3_, 45% K_2_O, 32% MgO, 45% Na_2_O, 40% P_2_O_5_, 50% SO_3_, 29% SiO_2_, 35% TiO_2_. When comparing the four analytical methods, note that a certain dispersion may also result from potential inhomogeneity of the samples. Although all analysed samples derived from the same basic population (dried and prepared samples), they were incinerated, digested and measured independently of each other.

**Table 5:** Minimum and maximum results as well as the relative deviation for all elemental oxides and analytical methods.

Sample Identification	Description of Result	Al_2_O_3_ [M.-%]	CaO [M.-%]	Fe_2_O_3_ [M.-%]	K_2_O [M.-%]	MgO [M.-%]	Na_2_O [M.-%]	P_2_O_5_ [M.-%]	SO_3_ [M.-%]	SiO_2_ [M.-%]	TiO_2_ [M.-%]	SUM [M.-%]
Primary 2	Minimum result	7.4	28.1	2.8	1.1	2.7	0.6	0.5	3.9	25.0	3.5	82.7
	Maximum result	8.6	32.9	3.8	3.1	4.3	3.5	1.0	5.6	30.2	4.2	92.5
	Relative deviation between minimum and maximum [%]	14.2	14.7	26.3	64.7	37.3	83.3	51.6	30.2	17.2	17.3	10.7
Primary 3	Minimum result	8.3	22.7	2.1	0.8	2.4	3.3	0.3	1.7	37.5	0.8	84.4
	Maximum result	10.3	25.0	3.3	2.1	3.6	3.8	0.4	4.2	43.7	1.3	92.4
	Relative deviation between minimum and maximum [%]	19.2	9.4	34.7	61.6	33.5	14.2	28.3	58.1	14.0	38.4	8.7
Primary 4	Minimum result	6.3	20.7	2.3	1.1	2.4	1.9	0.3	3.0	42.1	0.7	85.4
	Maximum result	7.6	22.2	3.5	1.8	3.5	3.4	0.6	4.4	48.7	1.0	93.8
	Relative deviation between minimum and maximum [%]	17.0	6.6	33.9	40.2	32.7	43.6	49.9	30.9	13.5	32.8	8.9
Primary 6	Minimum result	9.6	27.7	1.8	2.8	1.8	2.3	1.0	0.5	22.0	1.1	83.8
	Maximum result	11.6	42.4	3.0	5.4	3.1	3.5	2.9	5.6	29.5	1.6	104.4
	Relative deviation between minimum and maximum [%]	17.2	34.7	38.9	48.6	42.5	33.5	65.4	91.1	25.4	32.4	19.7
Primary 7	Minimum result	6.5	28.5	2.4	2.3	1.5	2.8	1.0	1.1	28.3	0.9	80.5
	Maximum result	10.1	33.8	3.8	3.0	3.2	4.5	1.6	5.7	31.9	1.5	92.0
	Relative deviation between minimum and maximum [%]	35.9	15.7	37.6	24.3	53.3	38.3	36.9	80.7	11.3	41.2	12.5
Secondary 17	Minimum result	11.4	28.3	3.3	0.7	2.7	1.1	1.0	0.2	25.6	1.6	86.5
	Maximum result	14.0	36.9	4.3	3.1	3.6	4.9	1.7	4.2	28.4	2.1	94.9
	Relative deviation between minimum and maximum [%]	18.5	23.3	23.2	77.7	25.0	78.4	42.9	95.0	10.0	24.8	8.8
Secondary 19	Minimum result	15.0	18.7	3.0	1.5	2.5	2.4	0.8	0.2	33.2	0.9	84.3
	Maximum result	19.2	28.1	3.9	3.0	3.5	4.7	1.0	4.5	35.9	1.7	99.2
	Relative deviation between minimum and maximum [%]	21.7	33.5	23.8	48.7	29.4	48.8	17.0	96.0	7.6	47.9	15.0
Secondary 20	Minimum result	6.5	20.0	2.8	1.5	2.4	2.5	0.3	2.8	43.6	0.8	90.6
	Maximum result	8.3	22.2	3.9	2.4	3.3	4.5	0.6	4.4	57.7	1.1	97.8
	Relative deviation between minimum and maximum [%]	22.2	9.9	29.5	36.7	27.7	43.6	55.2	37.5	24.5	28.5	7.4

A slightly higher relative deviation of 24.8 to 65.4% between the analytical methods was observed for TiO_2_, P_2_O_5_ and MgO. Even higher deviations between the different analytical methods were observed for K_2_O, Na_2_O and SO_3_. Most of the deviations were well above 40%. For the SO_3_ content, deviations between the four methods sometimes appear particularly significant. A possible explanation may be uncontrolled loss of sulphur, expressed as SO_2_, during sample preparation for fused beads since especially for methods including melting digestions, the results tend to be lower. Note that methods B and D (melting digestion and total digestion from the ash) generally display better agreement than each of them vs. XRF (method A) and method C (total digestion from the dried sample). Method C is generally considered less suitable for identifying main elements than the other analytical procedures described although it is one of the methods proposed in EN 15410 [38]. This is explained by the fact that only about 0.2 g of the dried and prepared (and non-incinerated) sample is used for total digestion. But only approx. 9.8 to 31.7% does actually constitute mineral matter (ash content; cf. Table 6) and is hence relevant for analysis. In other words, the sample fraction of 0.2 g examined and analysed is once more reduced, down to approx. 0.02 to 0.06 g. With such small sample quantities, any inhomogeneity included in the sample may severely impact the analytical result. In case a sample already incinerated is used for digestion, the observed sample has a quantity of at least 0.2 g, reducing potential impacts of inhomogeneity. This clearly emerged from evaluating the duplicate runs of each analytical method. For method C, the relative deviations between the duplicates were sometimes > 30%, for all other methods, they were < 10%.

**Table 6:** Total inorganic carbon content (TIC) and ash content for different ash samples in relation to the incineration temperature.

Sample identification	TIC Ash residue 550 °C [wt%]	TIC Ash residue 815 °C [wt%]	TIC Ash residue 950 °C [wt%]	Ash content 550 °C [wt%]	Ash content 815 °C [wt%]	Ash content 950 °C [wt%]
Primary 1	3.1	0.3	0.1	12.3	11.0	11.5
Primary 3	4.4	0.2	0.2	15.8	12.6	11.6
Primary 4	3.3	0.6	0.4	25.5	23.4	18.3
Primary 5	4.0	0.3	0.4	22.1	19.4	17.3
Primary 6	4.5	0.7	0.6	26.3	19.4	16.7
Primary 7	4.0	0.7	0.5	22.2	18.6	17.6
Primary 19	4.3	0.7	< 0.1	17.5	16.0	16.1
Primary 20	2.7	0.6	0.4	25.3	24.3	23.1
Primary 22	3.9	0.5	0.3	14.9	12.7	12.8
Primary 27	5.5	0.5	0.4	36.0	29.4	29.4
Primary 32	4.1	0.6	0.4	9.7	8.5	8.5
Primary 36	3.8	0.2	0.1	29.1	21.1	21.0
Primary 40	1.7	0.2	< 0.1	9.8	9.0	9.0
Primary 41	2.1	0.7	0.5	9.9	9.0	8.9
Primary 44	4.0	0.5	0.4	27.5	23.4	24.0
Secondary 17	3.1	0.4	0.5	15.2	13.4	13.2
Secondary 18	2.0	0.1	0.1	19.9	16.2	15.8
Secondary 19	4.0	0.6	0.8	18.9	17.0	17.2
Secondary 20	3.3	0.2	0.2	31.7	30.6	30.2
Secondary 21	1.7	0.2	0.1	16.1	12.3	12.8
Secondary 24	1.7	0.3	0.3	32.9	28.9	27.2
Secondary 28	4.1	0.6	0.4	19.2	16.5	15.2

### Effects of the incineration temperature on the R-index

The incineration temperature required for establishing the R-Index was an essential factor under discussion as the suggested approach was developed. 22 SRF samples (Primary 1, 3, 4, 5, 6, 7, 19, 20, 22, 27, 32, 36, 40, 41, 44 and Secondary 17, 18, 19, 20, 21, 24, 28) were incinerated at different temperatures, revealing distinct differences of the obtained ash content (see Table 6). The ash content obtained at 550 °C is much higher than any obtained at 815 °C or at 950 °C.

The EN 15403 [34] used for SRF stipulates an incineration temperature of 550 °C, however. This value is inappropriate for obtaining ash residue for subsequent digestion due to the following arguments:

• During a classical XRF investigation in cement manufacturing or geology, samples are incinerated before melting digestion, with incineration generally initiated at about 950 °C (cf. EN 196–2 [47]). Experimental procedures for XRF investigations (cf. Section 2.3) have shown that fused beads would repeatedly break or turn cloudy when residues incinerated from ashes at 550 °C were included. This has to be explained by the fact that e. g. carbonates may escape from the sample during melting expressed as CO_2_ so that they are not integrated into the structure of the fusion agent. That is why higher incineration temperatures should be applied to the production of ash residues.
• The DIN 51729–11 standard [40], intended for testing ashes from solid fuels, stipulates an incineration temperature of 815 °C in compliance with the DIN 51719 standard [35].
• A further argument supporting incineration temperatures of at least 815 °C and higher for analysing the ash content and to provide ash residue for subsequent main-element analysis is found in the fact that temperatures of about 1450 °C prevail in the rotary kilns of cement plants. So much heat can only be achieved on a laboratory scale with special equipment (kilns) while incineration temperatures of 800 °C to 1000 °C can regularly be achieved using conventional muffle furnaces, mimicking real conditions sufficiently well.
• When ashes are chemically analysed, the discovered elemental mass fractions are commonly converted into oxide mass fractions of the highest oxidation state, adding up to a total value of approx. 100 wt%. If this is not the case, then presumably either other components are present as well or the analytical procedure was incorrect. Consider further that the conversion of element concentrations (e. g. of Al) into oxides (e.g. Al_2_O_3_) is based on the assumption that all measured elements are indeed present as oxides. But for SRF it can be assumed that some elements (e.g. Ca, Mg, Fe) will be present as carbonates (e.g. calcium carbonate CaCO_3_, dolomite CaMg(CO_3_)_2_, siderite FeCO_3_). Thus, CaCO_3_ or CaMg(CO_3_)_2_ are not yet converted at an incineration temperature of 550 °C since the conversion into CaO does not initiate below approx. 800 °C. This means that only for incineration temperatures >800 °C, calcium etc. may be expected to be present as oxides with a high probability. Only incineration at suitably high temperatures helps making stoichiometric calculations of oxide contents accurate. If calcium would be given as calcium oxide though a part of it was expressed as carbonates, then the results would automatically be too low due to the much higher molar mass. The results of analysing the carbonate content (expressed as total inorganic carbon, TIC) (cf. Table 6) clearly show that residue incinerated at 550 °C still contains between 1.7 and 5.5 wt% of total inorganic carbon.

**Fig. 2:**
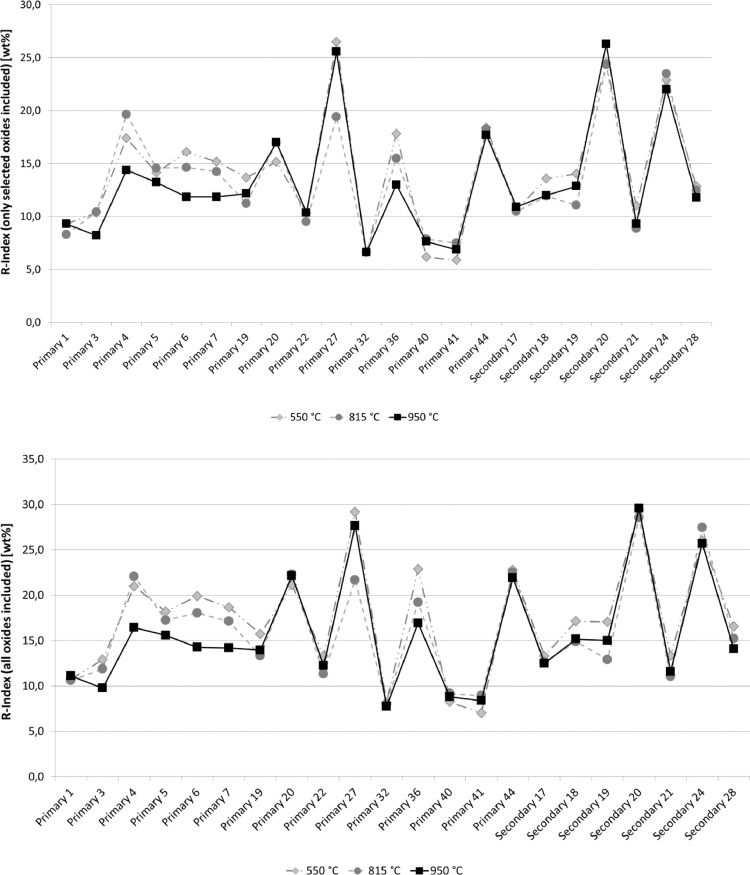
Comparison of the R-Indices for randomly selected SRF samples at different incineration temperatures; including all measured elementary oxides (above) as well as selected elementary oxides Al_2_O_3_, CaO, Fe_2_O_3_, SiO_2_ (below).

To compare the results for the 22 randomly selected SRF samples obtained at different incineration temperatures (550 °C, 815 °C and 915 °C), the R-Index was calculated using Formula 1 to directly relate to the respective ash content. At first, the total of all elementary oxides was used to calculate the R-index (cf. Fig. 2; above). Then, only a few selected elementary oxides (Al_2_O_3_, CaO, Fe_2_O_3_ and SiO_2_) were used to calculate the R-index (Fig. 2; below). For the ash content, the values for different incineration temperatures given in Table 6 were used in calculations.

Fig. 2 and Table A2 (cf. Appendices) clearly reveal that for different incineration temperatures, the differences between calculated R-Indices are negligible. The relative differences between the highest and lowest values of a sample are found between 0.1 and 26.7% (for calculations based on the selected elementary oxides Al_2_O_3_, CaO, Fe_2_O_3_ und SiO_2_) and between 4.6 and 26.0% (for calculations based on the total of all elementary oxides). This is because some elementary oxides are not yet present in the highest oxidation state when the incineration temperature is only 550 °C (say, Fe_2_O_3_, Al_2_O_3_), but this is not taken into account when the elementary oxide content is computed. On the other hand, some elements contained in the samples are still present as carbonates after incineration at 550 °C, which as well is not included in the conversion to elementary oxides. Therefore, computations incline to produce too low results for the total of all or for selected elementary oxides obtained at 550 °C vs. those values obtained at 815 °C or 950 °C (cf. Table A2). Multiplying this value with the higher ash content obtained at 550 °C, however, the result almost matches that of the R-Index at 950 °C or 815 °C.

The results in Fig. 2 and Table A2 clearly show that, while much may speak in favour of incineration temperatures higher than 815 °C, the incineration temperature does not have a major effect on the final result for the R-Index for most samples when referring the sum of oxides on the respective ash content obtained by different temperatures.

## Conclusions

Methods for finding the relative amount of recyclable SRF (known as the R-Index) have been presented in this paper. For this purpose, the total mineral content of SRF was classified as ash content and its main components were identified using various analytical methods. These methods are all based on available, though sometimes modified, standard methods. It was shown that all methods presented provide almost equivalent results (with the exception of Na_2_O, K_2_O and SO_3_). Neither the type of digestion (melt digestion, total digestion), the measurement method for determining the main mineral components (XRF, ICP-OES/ICP-MS) nor the incineration temperature (550 °C, 815 °C or 950 °C) significantly affect the final result obtained for the R-Index.

Essentially it was shown that using an already incinerated sample (in contrast to the dried and prepared sample) for digestion is recommendable to identifying the main mineral components. This is because any inhomogeneity may severely impact the result due to the low initial weight during digestion, impairing the accuracy of the analytical method. Moreover, the ash content of the proposed procedure for determining the R-Index has to be identified anyway, providing ash residue for subsequent main element analysis as a by-product.

The methods presented in this paper are all easy to implement particularly in laboratories already performing SRF or fuel analysis. The methods do not require special equipment but the necessary steps can be managed using established equipment. While in laboratories for SRF or fuel analysis wet chemistry methods seem to be more convenient, the company laboratory of cement plants generally using XRF instruments for quality assurance of raw materials and products, may as well apply available methods and instruments for obtaining the R-Index.

Based on the experiences gained during the experimental part of this paper, methods D and A can certainly be recommended as the most practical and most suitable approaches for determining the main components in SRF. Both methods deliver reliable results. From a technical perspective it is also recommended to apply incineration temperatures ≥ 815 °C for the determination of the ash content or providing the ash residue to be analysed.

Method D was also applied for the experimental investigations on 80 SRF samples currently on the market in Austria, Croatia, Slovakia, and Slovenia in regards to the determination of the material-recyclable share presented in the work of Viczek et al. 2020 [11].

The analytical methods developed and introduced in this paper allow for reliably calculating the R-index. The methods were extensively validated and their applicability in practice was evaluated. The application of these methods ensures that the results obtained on an international, practical, and scientific level are comparable and equivalent. This is the precondition to make generally valid statements about the material-recyclable share of SRF when co-processed in the cement industry. Determining this material-recyclable share is of major importance for the cement industry, waste treatment companies, and governmental institutions.

## Figures and Tables

**Table A1:** Results for the measured proficiency testing materials and the respective z-score.

Sample identification	Al_2_O_3_	CaO	Fe_2_O_3_	K_2_O	MgO	Na_2_O	P_2_O_5_	SO_3_	SiO_2_	TiO_2_	SUM
Proficiency testing material 1 [wt%]	26.3	25.5	3.3	1.5	2.1	1.6	0.9	2.5	27.6	3.7	94.9
**z-Score [-]**	**0.24**	**−0.14**	**−0.04**	**0.79**	**0.33**	**−0.78**	**0.09**	**0.80**	**0.92**	**−0.62**	**–**
Proficiency testing material 2 [wt%]	26.4	25.7	3.0	1.5	2.5	2.9	1.0	2.6	20.0	3.4	89.1
**z-Score [-]**	**−0.15**	**−0.13**	**−0.52**	**1.24**	**0.53**	**−0.50**	**0.11**	**0.71**	**0.14**	**−0.91**	**–**
Proficiency testing material 3 [wt%]	29.5	27.6	3.1	1.3	1.9	2.3	1.0	2.4	25.1	4.1	98.3
**z-Score [-]**	**−0.25**	**0.60**	**−0.03**	**0.76**	**−0.26**	**−1.85**	**0.61**	**1.02**	**0.67**	**0.16**	**–**

**Table A2:** Results for the R indices of randomly selected SRF samples at different incineration temperatures and considering all elemental oxides and selected elemental oxides (Al_2_O_3_, CaO, Fe_2_O_3_, SiO_2_).

Sample identification	R-Index (all elemental oxides included) [wt.%]			R-Index (only Al_2_O_3_, CaO, Fe_2_O_3_ and SiO_2_ included) [wt.%]		
	550 °C	815 °C	915 °C	550 °C	815 °C	915 °C
Primary 1	10.8	10.6	11.1	9.3	8.3	9.3
Primary 3	12.9	11.9	9.8	10.4	10.4	8.2
Primary 4	21.0	22.1	16.4	17.4	19.6	14.4
Primary 5	18.2	17.2	15.6	14.1	14.6	13.2
Primary 6	19.9	18.0	14.3	16.1	14.6	11.9
Primary 7	18.7	17.1	14.2	15.2	14.2	11.8
Primary 19	15.7	13.3	14.0	13.7	11.2	12.1
Primary 20	21.1	22.3	22.1	15.2	17.0	17.0
Primary 22	13.3	11.3	12.2	10.3	9.5	10.4
Primary 27	29.2	21.7	27.7	26.5	19.4	25.6
Primary 32	8.2	8.0	7.7	6.6	6.6	6.6
Primary 36	22.9	19.2	16.9	17.8	15.5	13.0
Primary 40	8.2	9.2	8.8	6.2	7.9	7.6
Primary 41	7.0	8.9	8.4	5.9	7.5	6.9
Primary 44	22.8	22.5	21.9	18.4	18.2	17.7
Secondary 17	13.3	12.6	12.5	10.6	10.5	10.9
Secondary 18	17.1	14.9	15.2	13.6	11.9	12.0
Secondary 19	17.1	12.9	15.0	14.0	11.1	12.9
Secondary 20	29.4	28.6	29.6	24.5	24.3	26.3
Secondary 21	13.3	11.1	11.6	11.0	8.8	9.3
Secondary 24	26.0	27.5	25.7	22.9	23.5	22.0
Secondary 28	16.5	15.2	14.1	12.8	12.5	11.8
